# Gene selection and classification for cancer microarray data based on machine learning and similarity measures

**DOI:** 10.1186/1471-2164-12-S5-S1

**Published:** 2011-12-23

**Authors:** Qingzhong Liu, Andrew H Sung, Zhongxue Chen, Jianzhong Liu, Lei Chen, Mengyu Qiao, Zhaohui Wang, Xudong Huang, Youping Deng

**Affiliations:** 1 Department of Computer Science, Sam Houston State University, Huntsville, TX 77341, USA; 2 Department of Computer Science and Institute of Complex Additive Systems Analysis, New Mexico Institute of Mining and Technology, Socorro, NM 87801, USA; 3 Biostatistics Epidemiology Research Design Core, Center for Clinical and Translational Sciences, The University of Texas Health Science Center at Houston, Houston, TX 77030, USA; 4 The Chem21 Group, Inc, 1780 Wilson Drive, Lake Forest, IL 60045, USA; 5 Mathematics and Computer Science, Dept. of Mathematics & Computer Science, South Dakota School of Mines & Technology, Rapid City, SD 57701-3995; 6 Wuhan University of Science and Technology, Wuhan, Hubei 430081, China; 7 Conjugate and Medicinal Chemistry Laboratory, Division of Nuclear Medicine and Molecular Imaging, Department of Radiology, Brigham and Women's Hospital and Harvard Medical School, Boston, MA 02115, USA; 8 Cancer Bioinformatics, Rush University Cancer Center, and Department of Internal Medicine, Rush University Medical Center, Chicago, IL 60612, USA

**Keywords:** gene selection, microarray, classification, supervised-learning, similarity

## Abstract

**Background:**

Microarray data have a high dimension of variables and a small sample size. In microarray data analyses, two important issues are how to choose genes, which provide reliable and good prediction for disease status, and how to determine the final gene set that is best for classification. Associations among genetic markers mean one can exploit information redundancy to potentially reduce classification cost in terms of time and money.

**Results:**

To deal with redundant information and improve classification, we propose a gene selection method, Recursive Feature Addition, which combines supervised learning and statistical similarity measures. To determine the final optimal gene set for prediction and classification, we propose an algorithm, Lagging Prediction Peephole Optimization. By using six benchmark microarray gene expression data sets, we compared Recursive Feature Addition with recently developed gene selection methods: Support Vector Machine Recursive Feature Elimination, Leave-One-Out Calculation Sequential Forward Selection and several others.

**Conclusions:**

On average, with the use of popular learning machines including Nearest Mean Scaled Classifier, Support Vector Machine, Naive Bayes Classifier and Random Forest, Recursive Feature Addition outperformed other methods. Our studies also showed that Lagging Prediction Peephole Optimization is superior to random strategy; Recursive Feature Addition with Lagging Prediction Peephole Optimization obtained better testing accuracies than the gene selection method varSelRF.

## Background

Using microarrays techniques, researchers can measure the expression levels for tens of thousands of genes in a single experiment to provide scientists functional relationship information between the cellular and physiological processes of biological organisms and genes at a genome-wide level. The preprocessing procedure for the raw microarray data consists of back-ground correction, normalization, and summarization. After preprocessing, high level analyses, such as gene selection, classification, or clustering, are executed for profiling gene expression patterns [1]. In the past decade, two main tracks of analyses of microarray data have been to partition genes into closely related groups across time using clustering techniques and to classify patients with different health statuses based on selected gene signatures [2-6]. Various standards related to systems biology are discussed by Brazma *et al. *[7]. When sample sizes are substantially smaller than the number of features/genes, statistical modeling and inference issues are challenging, which is known as the "large p small n problem". Two important questions and challenges for the high dimensional data analyses are how to choose features that provide reliable and good prediction and how to determine the final optimal feature set that is best for prediction and classification.

To address the "curse of dimensionality" problem, three strategies have been proposed: filtering, wrapper and embedded methods. Filtering methods select subset features independently from the learning classifiers and do not incorporate learning [8-11]. One of the weaknesses of filtering methods is that they only consider the individual features in isolation and ignore their possible interactions. Yet, the combination of these features may have a combined effect that does not necessarily follow from the individual performance of features in that group [12]. One of the consequences of filtering methods is that we may end up with many highly correlated features/genes; this highly redundant information will worsen classification and prediction performance. Furthermore, if there is a limit on the number of features to be chosen, we may not be able to include all informative features.

To avoid weakness in filtering methods, wrapper methods wrap around a particular learning algorithm that can assess the selected feature subsets in terms of estimated classification errors to build the final classifier [13]. Wrapper methods use a learning machine to measure the quality of subsets of features. One recent well-known wrapper method for feature/gene selection is Support Vector Machine Recursive Feature Elimination (SVMRFE), proposed by Guyon *et al. *[14], which refines the optimum feature set by using Support Vector Machine (SVM). The idea of SVMRFE is that the orientation of the separating hyper-plane found by the SVM can be used to select informative features: if the plane is orthogonal to a particular feature dimension, then that feature is informative, and vice versa. In addition to gene selection, SVMRFE has been successfully used in other feature selection and pattern classification situations [15,16].

Wrapper methods can noticeably reduce the number of features and significantly improve classification accuracy [17,18]. However, wrapper methods have the drawback of high computational load. With better computational efficiency and similar performance to wrapper methods, embedded methods process feature selection simultaneously with a learning classifier. Examples of embedded methods are LASSO [19,20] and logistic regression with the regularized Laplacian prior [21].

Combining the sequential forward selection (SFS) and sequential floating forward selection (SFFS) with LS (Least Squares) Bound measure, Zhou and Mao proposed SFS-LS bound and SFFS-LS bound algorithms for optimal gene selection [22]. Tang *et al*. also proposed two gene selection methods, leave-one-out calculation sequential forward selection (LOOCSFS) and the gradient based leave-one-out gene selection (GLGS) [23]. Diaz-Uriarte and De Andres [24] presented a new method for gene selection that uses random forest [25]. The main advantage of this method is that it returns very small sets of genes that retain high predictive accuracy. The algorithms are publicized in the R package of varSelRF. Additionally, Guyon and Elisseeff elaborated a wide range of aspects in feature selection including a better definition of the objective function, feature construction, feature ranking, multivariate feature selection, efficient search methods and feature validity assessment methods [26].

In human genetic research, exploiting information redundancy from highly correlated genes may potentially reduce the cost of classification in terms of time and money. To deal with redundancy issues and to improve classification for microarray data, we designed a gene selection method recursive feature addition (RFA) in our previous work [27], however, the optimal feature set associated with the best training was not solved. In this paper, we compare this method to SVMRFE, LOOCSFS, GLGS, SFS-LSbound, SFFS-LSbound and T-test by using six benchmark microarray data sets; meanwhile, we propose an algorithm, Lagging Prediction Peephole Optimization (LPPO), to choose the final optimal feature/gene set. We evaluate LPPO by comparing it with random strategy under the best training condition and valSelRF [24].

## Results

Under feature dimension *j*, the training accuracy of the *i*^th ^experiment is *r*(*i, j*), and the testing accuracy of the *i*^th ^experiment is *s*(*i, j*), *i *= 1, 2,..., *I*; *j *= 1, 2,..., *J*; where *I *is the number of experiments and *J *is the number of chosen features. The average testing accuracy of the experiments under the feature dimension *j*, *s*(*j*), *j *= 1, 2,..., *J*, is calculated as follows:

(1) $$s\left(j\right)=\frac{1}{I}\sum_{i=1}^{I}s\left(i,j\right)$$

The average testing accuracy, *ms_hr*(*i*), of the *i*^th ^experiment under the condition that the associated/corresponding training accuracy is the highest, which is defined as follows:

(2) $$ms\text{\_}hr\left(i\right)=\text{mean}\left(s\left(i,m\right)\right)\left|r\left(i,m\right)\right)=\max\left(r\left(i,j\right)\right),\forall m,j\in \left\{1,2,..J\right\}$$

The average testing accuracy *ms_hr*(*i*) is the expected value of the random strategy under the best training classification of the *i*^th ^experiment.

The highest testing accuracy, *hs_hr*(*i*), of the *i*^th ^experiment under the condition that the associated/corresponding training accuracy is the highest, which is defined as follows:

(3) hs_hr(i)=max(s(i,m))|r(i,m)= max(r(i,j)),∀m,j∈{1,2,..J}

### Average testing accuracy

**Figure 1:**
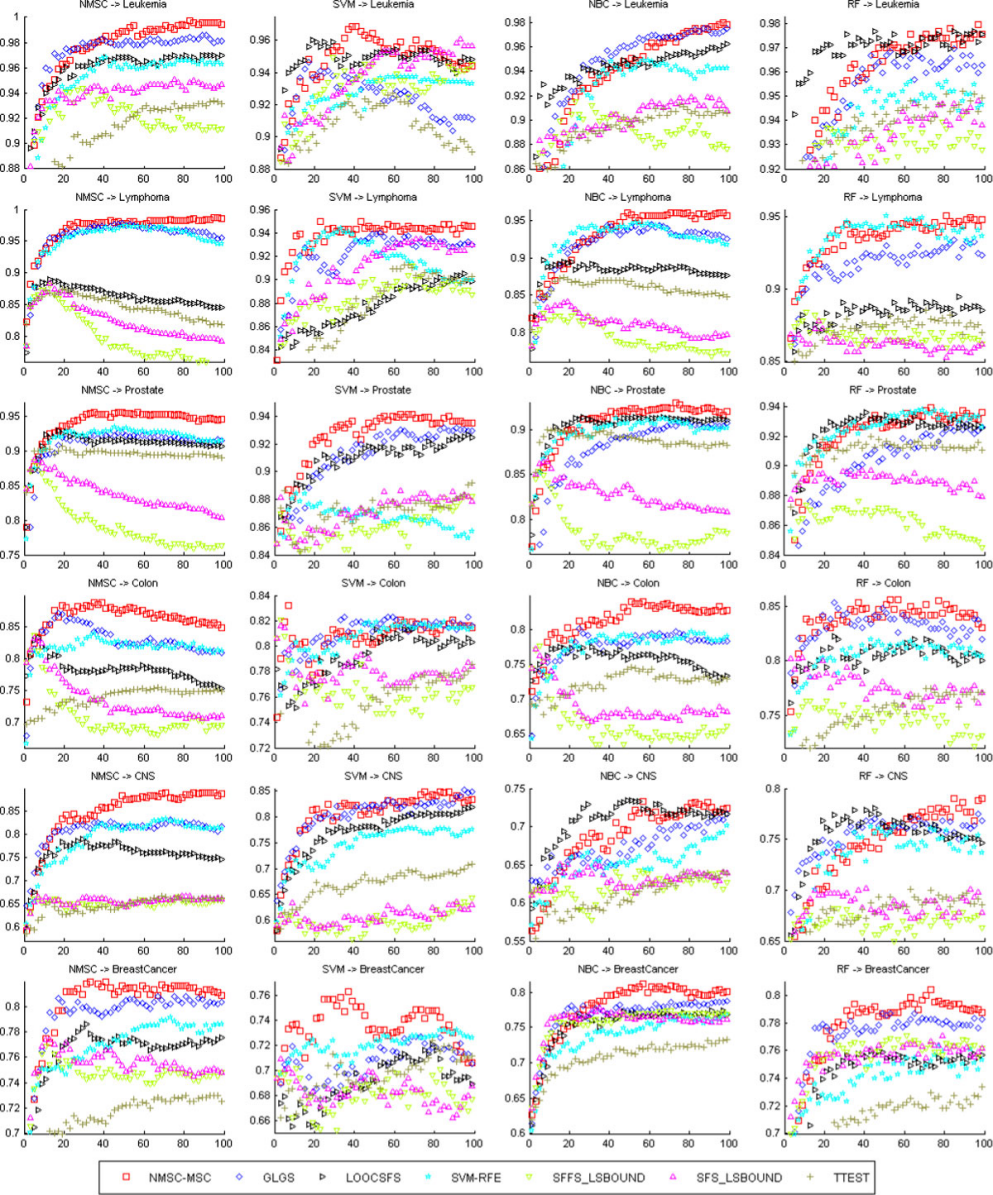
**The average testing accuracies of different gene selection methods for six benchmark data sets by using the classifiers (NBC, NMSC, SVM, RF)**. X-axis and y-axis give the feature dimension and testing accuracy values, respectively.

Figure 1 lists the average testing accuracies of the gene selection methods with classifiers NMSC, SVM, NBC, and RF. Again, the performances of NBC-MMC, NMSC-MMC, NBC-MSC, and NMSC-MSC are close to one another; therefore, the average testing accuracies of the gene selection methods NBC-MMC, NMSC-MMC, and NBC-MSC are not listed in the figures. It indicates that the average testing accuracy of NMSC-MSC is the best, followed by GLGS, LOOCSFS, and SVM-RFE. SFS-LS bound, SFFS-LS bound, and T-TEST did not perform well. Figure 1 also demonstrates that, spanning several data sets and learning classifiers, the performance and stabilization of the gene selection method of NMSC-MSC is the best.

### Testing results under the best training

Table 1 provides the mean values and standard errors of the testing accuracies ms_hr(i), (i = 1, 2,..., 20) and the highest testing accuracies hs_hr(i), (i = 1, 2, ..., 20) under the highest training classification, respectively. After applying each classifier to each data set, the highest mean value of the ten gene selection methods is shaded. In each data set, the highest mean value in the shade is in bold. With the use of the four learning classifiers, under the best training, RFA, GLGS, LOOCSFS, SVMRFE, SFS-LSBOUND, SFFS-LSBOUND, and T-test respectively achieve the highest testing accuracies (HS_HR), 99.9%, 99.6%, 99.3%, 98.0%, 97.4%, 97.3%, and 96.8% for the leukemia data set; 99.5%, 98.6%, 93.0%, 99.2%, 95.1%, 96.1%, and 94.4% for lymphoma; 96.9%, 96.1%, 95.2%, 95.7%, 93.4%, 92.7%, and 94.0% for prostate; 91.1%, 90.5%, 86.8%, 86.8%, 87.1%, 86.0%, and 85.5% for colon; 94.0%, 91.1%, 85.0%, 85.1%, 76.2%, 76.2%, and 77.4% for CNS; and 85.9%, 83.7%, 80.3%, 80.4%, 81.5%, 81.3%, and 77.6% for the breast cancer data set. In applying the ten gene selection methods to the six benchmark data sets, all the highest testing accuracies are obtained from the gene set chosen by RFA.

**Table 1:** Mean values and standard errors of hs_hr and ms_hr.

DATA SET	GENE SELECTION METHOD	MEAN(HS_HR) ± STD(HS_HR), %				MEAN(MS_HR) ± STD(MS_HR), %			
		
		NMSC	SVM	NBC	RF	NMSC	SVM	NBC	RF
Leukemia	NBC-MMC	**99.9 ± 0.6**	99.4 ± 1.2	98.3 ± 2.3	98.4 ± 1.4	98.1 ± 1.4	93.4 ± 2.8	94.3 ± 2.8	95.6 ± 2.3
	NMSC-MMC	**99.9 ± 0.6**	99.1 ± 1.3	98.4 ± 1.9	98.6 ± 1.9	97.9 ± 1.2	93.3 ± 2.8	95.2 ± 2.8	95.7 ± 3.4
	NBC-MSC	99.4 ± 1.1	99.1 ± 1.3	**98.9 ± 1.4**	98.4 ± 1.7	**98.5 ± 1.6**	**94.9 ± 2.7**	94.6 ± 2.7	96.0 ± 2.5
	NMSC-MSC	99.7 ± 0.9	**99.6 ± 1.0**	98.6 ± 1.7	98.7 ± 1.7	97.7 ± 1.4	94.8 ± 2.5	94.6 ± 3.4	95.7 ± 3.1
	GLGS	99.6 ± 1.0	98.9 ± 1.7	98.6 ± 1.7	98.6 ± 1.7	97.8 ± 1.7	92.5 ± 3.8	**95.3 ± 1.8**	95.0 ± 2.5
	LOOCSFS	97.1 ± 3.3	98.0 ± 1.5	97.7 ± 1.9	**99.3 ± 1.2**	93.9 ± 3.5	94.8 ± 3.1	94.5 ± 2.7	**96.7 ± 1.6**
	SVMRFE	98.0 ± 2.0	95.4 ± 3.9	97.3 ± 2.1	98.0 ± 2.0	95.7 ± 2.8	92.5 ± 5.2	92.5 ± 3.0	93.4 ± 1.9
	SFFS-LSBOUND	97.1 ± 2.5	97.4 ± 3.8	96.3 ± 4.1	97.1 ± 2.8	93.8 ± 4.3	92.9 ± 3.8	90.2 ± 5.8	92.6 ± 4.1
	SFS-LSBOUND	97.1 ± 2.8	97.0 ± 3.0	96.4 ± 3.6	97.3 ± 3.0	94.6 ± 3.5	93.6 ± 3.8	91.2 ± 5.0	93.0 ± 5.1
	T-TEST	94.8 ± 3.5	95.4 ± 4.5	93.3 ± 6.9	96.8 ± 2.9	92.2 ± 3.9	90.7 ± 4.8	90.1 ± 6.5	93.5 ± 3.6

Lymphoma	NBC-MMC	98.1 ± 2.6	**99.0 ± 1.3**	97.3 ± 2.6	96.4 ± 2.8	96.2 ± 4.3	93.8 ± 2.8	91.7 ± 3.9	91.6 ± 3.7
	NMSC-MMC	99.2 ± 1.2	98.8 ± 1.6	97.9 ± 2.6	96.5 ± 3.7	96.9 ± 1.9	93.0 ± 2.8	93.1 ± 3.3	92.3 ± 4.0
	NBC-MSC	99.4 ± 1.1	98.4 ± 1.8	97.9 ± 2.6	96.8 ± 3.3	**97.5 ± 1.9**	93.1 ± 3.5	92.7 ± 3.5	92.6 ± 4.1
	NMSC-MSC	**99.5 ± 1.1**	98.8 ± 1.6	**98.1 ± 2.0**	**97.0 ± 3.6**	97.2 ± 1.9	**93.9 ± 3.0**	**93.9 ± 3.1**	**93.4 ± 3.9**
	GLGS	98.6 ± 1.8	98.2 ± 1.9	97.0 ± 2.6	96.9 ± 2.3	96.5 ± 2.1	92.5 ± 3.8	92.3 ± 3.6	91.7 ± 2.9
	LOOCSFS	87.0 ± 7.2	93.0 ± 5.3	87.3 ± 5.1	92.9 ± 4.8	85.8 ± 6.8	87.8 ± 5.4	85.1 ± 4.5	88.2 ± 4.3
	SVMRFE	99.2 ± 1.5	96.5 ± 3.9	97.2 ± 3.4	96.6 ± 3.1	96.5 ± 2.0	91.8 ± 4.3	93.1 ± 4.0	93.3 ± 4.0
	SFFS-LSBOUND	88.7 ± 6.1	95.1 ± 3.3	84.0 ± 4.9	92.2 ± 4.7	87.0 ± 5.7	88.2 ± 4.9	80.6 ± 3.9	86.8 ± 4.8
	SFS-LSBOUND	87.7 ± 6.1	96.1 ± 3.5	86.1 ± 3.5	91.8 ± 4.2	86.4 ± 5.6	91.1 ± 3.7	82.7 ± 3.4	86.1 ± 4.8
	T-TEST	86.0 ± 5.7	94.4 ± 3.0	86.5 ± 7.0	91.7 ± 5.2	84.3 ± 5.8	87.7 ± 3.3	83.9 ± 6.1	87.2 ± 4.5

Prostate	NBC-MMC	96.3 ± 2.4	95.8 ± 2.5	94.8 ± 2.6	**96.5 ± 2.0**	94.2 ± 2.8	91.6 ± 2.3	90.4 ± 2.7	92.1 ± 2.2
	NMSC-MMC	95.6 ± 2.3	95.9 ± 2.5	93.7 ± 2.8	95.3 ± 2.3	92.7 ± 2.3	91.4 ± 2.8	90.7 ± 3.1	91.3 ± 2.3
	NBC-MSC	96.4 ± 2.0	96.6 ± 1.9	**95.2 ± 2.1**	**96.5 ± 1.9**	**94.6 ± 2.3**	92.5 ± 2.3	91.0 ± 2.3	**92.5 ± 2.2**
	NMSC-MSC	**96.9 ± 2.3**	**96.7 ± 1.7**	94.5 ± 2.0	95.8 ± 1.8	94.5 ± 2.4	**92.8 ± 1.9**	**91.8 ± 2.5**	92.0 ± 1.9
	GLGS	93.6 ± 3.0	96.1 ± 2.2	90.4 ± 3.9	94.7 ± 2.0	91.5 ± 2.7	91.7 ± 2.6	87.5 ± 3.4	90.0 ± 2.5
	LOOCSFS	88.4 ± 5.2	94.9 ± 2.9	90.7 ± 5.3	95.2 ± 2.6	87.0 ± 4.7	91.1 ± 3.4	88.0 ± 4.5	92.3 ± 2.3
	SVMRFE	94.1 ± 3.4	92.3 ± 2.7	92.8 ± 4.3	95.7 ± 2.6	92.4 ± 3.3	86.7 ± 3.5	90.0 ± 4.0	**92.5 ± 2.8**
	SFFS-LSBOUND	90.4 ± 3.2	93.4 ± 2.8	86.2 ± 5.8	90.2 ± 3.2	88.9 ± 3.1	86.0 ± 3.2	84.4 ± 5.1	86.1 ± 4.0
	SFS-LSBOUND	89.7 ± 4.9	92.7 ± 4.0	87.3 ± 5.4	92.4 ± 3.5	88.3 ± 5.1	87.2 ± 5.0	85.1 ± 5.4	89.0 ± 3.9
	T-TEST	91.4 ± 4.1	92.5 ± 2.1	91.7 ± 2.8	94.0 ± 3.0	89.7 ± 3.7	87.1 ± 3.2	89.0 ± 4.3	91.0 ± 3.1

Colon	NBC-MMC	88.7 ± 5.5	**87.7 ± 5.2**	86.5 ± 4.0	89.7 ± 4.9	84.5 ± 5.2	80.9 ± 6.0	78.2 ± 4.9	82.5 ± 5.5
	NMSC-MMC	**91.1 ± 5.0**	**87.7 ± 3.9**	87.4 ± 5.3	90.0 ± 4.0	84.9 ± 7.1	**81.3 ± 5.5**	80.8 ± 5.9	83.3 ± 5.4
	NBC-MSC	89.4 ± 4.3	86.9 ± 4.6	**88.7 ± 6.0**	90.0 ± 4.0	**86.0 ± 5.2**	80.3 ± 5.6	82.1 ± 4.8	**84.4 ± 4.7**
	NMSC-MSC	91.0 ± 5.3	87.6 ± 4.7	88.1 ± 3.3	90.0 ± 4.4	**86.0 ± 5.4**	80.9 ± 5.5	**82.6 ± 4.0**	83.9 ± 4.5
	GLGS	87.3 ± 6.2	87.3 ± 4.6	85.2 ± 4.8	**90.5 ± 4.3**	83.7 ± 6.6	81.2 ± 5.5	77.6 ± 5.8	83.0 ± 4.5
	LOOCSFS	85.0 ± 5.3	86.3 ± 3.9	81.6 ± 5.8	86.8 ± 5.3	82.2 ± 4.6	79.3 ± 5.2	76.7 ± 6.9	80.3 ± 5.3
	SVMRFE	86.0 ± 6.7	86.8 ± 4.8	82.1 ± 7.4	86.3 ± 5.5	81.8 ± 7.2	80.7 ± 4.7	77.7 ± 7.5	80.3 ± 6.0
	SFFS-LSBOUND	85.0 ± 4.8	87.1 ± 4.4	72.7 ± 7.0	82.6 ± 6.0	82.4 ± 4.4	76.2 ± 6.3	69.5 ± 8.3	74.6 ± 6.8
	SFS-LSBOUND	85.3 ± 4.6	85.8 ± 5.3	76.8 ± 7.1	86.0 ± 4.1	83.3 ± 4.7	77.7 ± 6.4	72.5 ± 6.2	77.6 ± 4.5
	T-TEST	77.4 ± 10.4	85.5 ± 4.0	76.3 ± 8.3	81.5 ± 7.2	74.9 ± 10.8	75.3 ± 5.7	72.8 ± 8.2	75.1 ± 7.8

CNS	NBC-MMC	91.8 ± 6.1	**92.9 ± 3.6**	77.8 ± 5.2	**85.7 ± 4.0**	86.7 ± 6.0	82.4 ± 4.7	67.3 ± 4.1	**76.3 ± 4.0**
	NMSC-MMC	90.0 ± 6.4	92.2 ± 5.7	78.0 ± 5.3	82.7 ± 5.2	82.8 ± 6.8	82.1 ± 5.6	67.5 ± 5.5	73.5 ± 4.9
	NBC-MSC	**94.0 ± 4.6**	92.0 ± 4.4	81.1 ± 4.1	85.5 ± 4.9	**88.4 ± 5.2**	**82.6 ± 5.5**	70.2 ± 3.7	75.9 ± 5.3
	NMSC-MSC	92.8 ± 4.0	91.6 ± 4.9	**81.3 ± 6.1**	84.9 ± 4.1	85.6 ± 4.3	81.4 ± 6.2	70.0 ± 4.5	74.4 ± 4.2
	GLGS	84.7 ± 3.3	91.1 ± 5.4	78.8 ± 5.5	84.2 ± 5.0	82.4 ± 3.6	81.3 ± 4.8	67.9 ± 4.5	75.3 ± 4.3
	LOOCSFS	71.3 ± 9.8	85.0 ± 5.9	79.1 ± 7.7	83.2 ± 4.4	69.3 ± 8.0	77.6 ± 4.5	**71.8 ± 6.2**	75.3 ± 5.1
	SVMRFE	83.2 ± 8.9	85.1 ± 8.4	77.1 ± 6.8	83.5 ± 4.3	77.0 ± 8.0	75.0 ± 8.8	65.7 ± 7.2	73.3 ± 4.9
	SFFS-LSBOUND	68.1 ± 6.7	71.9 ± 7.1	67.6 ± 7.7	76.2 ± 4.5	65.3 ± 6.3	59.4 ± 7.5	61.3 ± 6.1	66.9 ± 4.8
	SFS-LSBOUND	67.8 ± 6.2	72.4 ± 4.9	69.8 ± 8.2	76.2 ± 5.0	65.7 ± 5.4	60.7 ± 5.1	63.7 ± 7.2	68.4 ± 4.5
	T-TEST	67.5 ± 8.8	77.4 ± 6.4	67.0 ± 7.1	75.5 ± 5.9	63.4 ± 7.6	67.3 ± 5.8	60.9 ± 6.8	67.8 ± 4.9

Breast	NBC-MMC	82.5 ± 6.0	82.9 ± 3.5	84.1 ± 3.0	84.1 ± 3.6	81.3 ± 5.7	73.2 ± 3.8	78.4 ± 3.4	78.4 ± 3.8
	NMSC-MMC	**83.9 ± 4.6**	82.0 ± 3.3	82.4 ± 4.3	83.7 ± 4.7	80.4 ± 4.0	72.0 ± 3.8	78.4 ± 4.3	77.0 ± 4.3
	NBC-MSC	83.4 ± 5.8	**83.5 ± 3.8**	**85.8 ± 3.1**	**85.9 ± 4.7**	**81.5 ± 5.3**	**74.9 ± 3.3**	79.1 ± 3.0	**79.4 ± 4.1**
	NMSC-MSC	82.8 ± 4.4	82.4 ± 3.8	84.1 ± 4.0	83.9 ± 4.0	79.6 ± 4.0	73.7 ± 3.9	**79.2 ± 3.8**	77.7 ± 4.0
	GLGS	80.8 ± 3.7	79.3 ± 4.5	81.4 ± 4.1	83.7 ± 4.6	79.2 ± 3.9	70.7 ± 4.6	77.8 ± 3.7	77.0 ± 4.2
	LOOCSFS	71.7 ± 6.5	77.3 ± 5.2	78.0 ± 5.8	80.3 ± 3.8	70.4 ± 6.5	69.2 ± 4.7	74.7 ± 5.1	74.3 ± 4.2
	SVMRFE	74.3 ± 7.1	78.3 ± 5.2	77.2 ± 5.3	80.4 ± 4.1	73.2 ± 6.6	72.1 ± 5.8	73.9 ± 4.5	73.9 ± 3.7
	SFFS-LSBOUND	76.2 ± 5.2	78.9 ± 2.8	76.9 ± 7.3	81.5 ± 5.3	75.0 ± 5.3	67.8 ± 3.3	75.2 ± 6.8	75.6 ± 4.9
	SFS-LSBOUND	77.5 ± 5.6	78.9 ± 4.2	79.8 ± 5.2	81.3 ± 5.2	75.8 ± 5.5	68.0 ± 4.7	76.9 ± 6.3	75.4 ± 5.2
	T-TEST	71.1 ± 5.3	77.6 ± 5.2	72.6 ± 6.3	76.3 ± 5.7	69.3 ± 5.3	69.9 ± 3.6	70.5 ± 5.8	71.1 ± 5.8

In each data set, the highest mean value is highlighted in bold

Table 2 lists the number of occurrences for each gene selection method that achieved the best testing accuracy. Table 2 shows that 61 out of 67 highest mean values were obtained by MMC- or MSC-based methods; GLGS, LOOCSFS, and SVMRFE obtained the best twice, three times, and once, respectively; LSBOUND and T-TEST never got the best value. Results indicate that RFA outperforms other gene selection methods.

**Table 2:** The number of occurrences of the best testing in Table 1

Gene Selection	# Best testing accumulated with each classifier		# Best testing among the four classifiers	
	
	HS_HR	MS_HR	HS_HR	MS_HR
NBC-MMC	6	1	1	0
NMSC-MMC	4	1	2	0
NBC-MSC	8	12	2	6
NMSC-MSC	7	8	2	1
GLGS	1	1	0	0
LOOCSFS	1	2	0	0
SVMRFE	0	1	0	0
SFFS-LSBOUND	0	0	0	0
SFS-LSBOUND	0	0	0	0
T-TEST	0	0	0	0
Total	27	26	7	7

On the other side, to see whether the new methods are superior to others, regression models were built based on average testing accuracy (ms_hr) and highest testing accuracy (hs_hr), respectively, with data set (six benchmark microarray data set), gene selection method (four new methods and six other methods) and classifier (four classification methods) as independent variables. After adjusting data set effect and classifier effect, the main effects for the new feature selection methods (NBC-MMC, NMSC-MMC, NBC-MSC, and NMSC-MSC) and others (GLGS, LOOCSFS, SVMRFE, SFS-LSBOUND, SFFS-LSBOUND, and T-test) are 91.86%, 91.67%, 92.47%, 92.27%, 90.65%, 86.96%, 88.89%, 84.70%, 85.38%, and 83.93% for the highest testing accuracy, and 86.38%, 86.15%, 87.30%, 86.97%, 85.48%, 82.76%, 83.96%, 79.45%, 80.58%, and 79.36% for the average testing accuracy, respectively. Table 3 gives the p-values of testing superiority of each new method to other six methods, which are calculated based on one-tailed t-test from the output of the regression models. From the p-values, the performances of our new methods are statistically significantly better than all other methods (most p-values are <0.0001) except for GLGS. From Table 3 MSC-based methods (NBC-MSC, NMSC-MSC) are significantly better than GLGS based on both highest testing accuracy and average testing accuracy at a significance level of 0.05. Although the p-values for NBC-MMC and NMSC-MMC to GLGS are not small enough due to the small sample size (only six testing data sets) and therefore lower power, we would expect that the differences will be detected at lower significance levels if more data sets are used. To see whether the four new gene methods perform differently, we also test each pair of the four methods and calculate the p-values based on two-tailed t-test from the output of the regression models. All the p-values are bigger than 0.2, so the four new methods perform equally well.

**Table 3:** P-values from testing superiority of new methods to others

Method	NBC-MMC		NMSC-MMC		NBC-MSC		NMSC-MSC	
	
	HS_HR	MS_HR	HS_HR	MS_HR	HS_HR	MS_HR	HS_HR	MS_HR
GLGS	0.092	0.15	0.13	0.22	0.023	0.0212	0.038	0.048
LOOCSFS	<0.0001	<0.0001	<0.0001	0.0001	<0.0001	<0.0001	<0.0001	<0.0001
SVMRFE	<0.0001	<0.0001	<0.0001	0.0077	<0.0001	0.0001	<0.0001	0.0004
SFFS-LSBOUND	<0.0001	<0.0001	<0.0001	<0.0001	<0.0001	<0.0001	<0.0001	<0.0001
SFS-LSBOUND	<0.0001	<0.0001	<0.0001	<0.0001	<0.0001	<0.0001	<0.0001	<0.0001
T-TEST	<0.0001	<0.0001	<0.0001	<0.0001	<0.0001	<0.0001	<0.0001	<0.0001

### Comparison of LPPO and random strategy

Table 4 lists the mean values of the differences between the testing values (denoted as S_LPPO) by applying NMSC, SVM, NBC, and RF to LPPO and ms_hr. This table shows that, on average, LPPO is superior to the random strategy under the best training accuracies. In summary, spanning the six benchmark data sets, in comparison with ms_hr, LPPO improves the testing accuracy by 0.8% for NMSC, 0.7% for SVM, 0.4% for NBC, and 0.9% for RF on average.

**Table 4:** Comparison of LPPO and Random Strategy

Data Set	Gene Selection	MEAN(S_LPPO - MS_HR), %			
		
		NMSC	SVM	NBC	RF
Leukemia	NBC-MMC	0.8	-0.1	2.3	1.4
	NMSC-MMC	1.0	0.9	1.8	1.6
	NBC-MSC	-0.2	0.3	1.9	1.1
	NMSC-MSC	1.6	0.7	2.5	1.3

Lymphoma	NBC-MMC	0.6	0.1	-1.0	0.4
	NMSC-MMC	1.3	-0.4	1.4	1.2
	NBC-MSC	0.4	1.2	1.5	1.4
	NMSC-MSC	0.9	0.1	1.6	0.6

Prostate	NBC-MMC	0.2	0.1	0.0	0.5
	NMSC-MMC	0.9	0.4	0.9	1.1
	NBC-MSC	0.3	0.7	0.6	1.8
	NMSC-MSC	0.4	0.8	0.2	1.0

Colon	NBC-MMC	0.3	0.2	-1.1	0.4
	NMSC-MMC	0.6	0.0	0.1	0.3
	NBC-MSC	-0.2	-0.5	-2.6	-1.3
	NMSC-MSC	0.9	0.3	-2.2	-0.5

CNS	NBC-MMC	2.1	1.8	2.2	3.1
	NMSC-MMC	0.8	1.0	0.4	1.6
	NBC-MSC	1.2	0.0	0.6	0.6
	NMSC-MSC	1.9	2.2	2.4	1.3

Breast Cancer	NBC-MMC	0.2	1.3	0.5	1.5
	NMSC-MMC	0.6	3.2	-1.2	0.9
	NBC-MSC	0.0	1.7	-1.6	-0.6
	NMSC-MSC	1.7	1.3	-1.1	1.0

Average		0.8	0.7	0.4	0.9

### Comparison of LPPO and varSelRF

**Figure 2:**
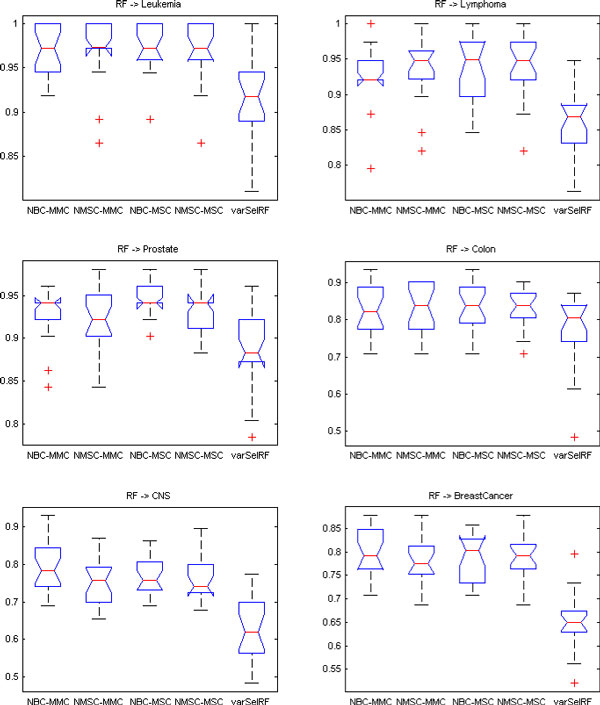
**Boxplots of testing accuracies of the LPPO with four gene selection methods using two different classifiers (NBC, NMSC) compared to varSelRF for six data sets**. RF is the final classifier. All six data sets demonstrate that varSelRF accuracies are lower than our proposed feature selection and optimization algorithm with the same RF classifier.

Figure 2 gives the boxplots of the testing values with the use of learning classifier random forest for the feature sets from LPPO with RFA and varSelRF. The gene selection methods are NBC-MMC, NMSC-MMC, NBC-MSC, NMSC-MSC, and varSelRF from left to right in each subfigure. Figure 2 indicates that the testing accuracies by applying random forest to the feature sets of LPPO with RFA are better than those of varSelRF. In comparison with varSelRF, LPPO with RFA increases the average testing accuracy by about 5% for the leukemia data set, 9% for lymphoma, 3% for colon and prostate, 10% for CNS, and 14% for the breast cancer data set.

### Computational efficiency

In microarray data analysis, generally, the number of features in the final feature set is far smaller than the total variables. Suppose the number of total variables is *n*, the number of features of the final feature set is *m *(*m *<<*n*). In forward feature selection, with the use of some learning classifier, the computational time is *F*(*s*, *d*) for a *s*×*d *feature matrix, here *s *is the number of data samples (s << n) and *d *is the feature dimensionality at each sample. Without losing the generality, if *d*_1_<*d*_2_, *F*(*s*, *d*_1_) <*F*(*s*, *d*_2_). The computational cost of our feature selection algorithm is analyzed as follows.

Let T_1 _denote the total computational time for supervised learning

(4) $$\begin{array}{ccc} T_{1} & =\;n*F\left(s,1\right)\;+\left(n-1\right)*F\left(s,2\right)\;+\;\ldots \;+\;\left(n-m+1\right)*F\left(s,m\right) & \\ \leq \left[n+\left(n-1\right)+...+\left(n-m+1\right)\right]*F\left(s,m\right) & \\ =\frac{m*\left(2*n-m+1\right)}{2}*F\left(s,m\right) & \end{array}$$

Let T_2 _denote the computational time for similarity calculation among the candidates and chosen genes, the calculation time between two single- variant vectors with s samples is C(s), then

(5) $$\begin{array}{ccc} T_{2} & \leq \left(n-1\right)*C\left(s\right)+2*\left(n-2\right)*C\left(s\right)+...+m*\left(n-m\right)*C\left(s\right) & \\ =C\left(s\right)*\left\{\frac{1}{2}nm\left(m+1\right)-\overset{m}{\underset{i=1}{\sum }}i^{2}\right\} & \end{array}$$

Due to the fact of m << n and s << n with microarray data, the computational cost of our feature selection is obtained by

(6) $$T=T_{\text{1}}+T_{2}\sim O\left(n\right)$$

## Conclusions

Our study shows that our gene selection method Recursive Feature Addition (RFA) obtained the best classification performance in the comparison. RFA utilizes supervised learning to obtain the best classification, and indentifies the subsequent gene recursively based on the similarity measures between the chosen gene set and the candidates to minimize the redundancy of the genes within the selected subset; hence it obtains more informative and differently expressed genes. Based on RFA, we also propose an algorithm, Lagging Prediction Peephole Optimization (LPPO), to determine the optimal feature set. Using six popular benchmark data sets, we compared RFA with other gene selection methods. Our studies showed that RFA outperformed other methods with the use of the four popular classifiers: NMSC, NBC, SVM, and random forest. Results also showed that, on average, LPPO is superior to a random strategy under the best training and that it outperformed the random forest based gene selection method varSelRF.

## Methods

### Supervised recursive learning

Our method of RFA uses supervised learning to achieve the highest level of training accuracy and statistical similarity measures to choose the next variable with the least dependence on or correlation to the already identified variables as follows:

1. Insignificant genes are removed according to their statistical insignificance. Specifically, a gene with a high p-value is usually not differently expressed and therefore has little contribution in distinguishing normal tissues from tumor tissues or in classifying different types of tissues. To reduce the computational load, those genes should be removed. The filtered gene data is then normalized. Here we use the standard normalization method, MANORM, which is available from MATLAB bioinformatics toolbox.

2. Each individual gene is selected by supervised learning. A gene with highest classification accuracy is chosen as the most important feature and the first element of the feature set. If multiple genes achieve the same highest classification accuracy, the one with the lowest *p*-value measured by test-statistics (e.g., score test), is the target of the first element. At this point the chosen feature set, **G**_1_, contains just one element, *g*_1_, corresponding to the feature dimension one.

3. The (*N*+1)^st ^dimension feature set, **G**_*N*+1 _= {*g*_1, _*g*_2_, ..., *g_N_*, *g*_*N*+1_} is obtained by adding *g*_*N*+1 _to the *N*^*th *^dimension feature set, **G**_*N *_= {*g*_1, _*g*_2_, ..., *g_N_*}. The choice of *g*_*N*+1 _is described as follows:

Add each gene *g_i _*(*g_i _*∉ **G***_N_*) into **G***_N _*and obtain the classification accuracy of the feature set **G***_N _*∪{*g_i_*}. The *g_i _*(*g_i _*∉ **G***_N_*) associated with the group, **G***_N _*∪{*g_i_*} that obtains the highest classification accuracy, is the candidate for *g*_*N*+1 _(not yet *g*_*N*+1_). Considering the large number of variables, it is highly possible that multiple features correspond to the same highest classification accuracy. These multiple candidates are placed into the set **C**, but only one candidate from **C **will be identified as *g*_*N+1*_. How to make the selection is described next.

### Candidate feature addition

To find the most informative (or least redundant) next feature *g*_*N*+1_, two formulas may be designed by measuring the statistical similarity between the chosen feature set and each candidate. Here we use, say, Pearson's correlation coefficient [28] between chosen features *g_n _*(*g_n _*∈ **G***_N _, n = *1, 2, *..*., *N*) and candidate *g_c _*(*g_c _*∈ **C**) to measure the similarity.

In the first formula, the sum of the square of the correlation, SC, is calculated to measure the similarity and is defined as follows:

(7) $$\text{SC}\left(g_{c}\right)=\overset{N}{\underset{n=1}{\sum }}\text{co}{\text{r}}^{\text{2}}\left(g_{c},\;g_{n}\right)\;\;n=1,2\;...N$$

Where, *g_c _*∈ **C***, g_n _*∈ **G***_N_*.

Then selection of *g*_*N*+1 _can be based on the Minimum Sum of the square of the Correlation (MSC), that is,

(8) $$g_{N+1}\leftarrow \left\{g_{c}|\text{SC}\left(g_{c}\right)=\min\left(\text{SC}\right).g_{c}\in C\right\}$$

In the second formula, the maximum value of the square of the correlation, MC, is calculated:

(9) $$\text{MC}\left(g_{c}\right)=\max\left(\text{co}{\text{r}}^{2}\left(g_{c},g_{n}\right)\right),\;\;n=1,2,...,N$$

Where, *g_c _*∈ **C***, g_n _*∈ **G***_N_*.

The selection of *g_N+1 _*follows the criterion that the MC value is the minimum, which we call Minimum of Maximum value of the square of the Correlation (MMC).

(10) $$g_{N+1}\leftarrow \left\{g_{c}|\text{MC}\left(g_{c}\right)=\min\left(\text{MC}\right).g_{c}\in C\right\}$$

In the methods mentioned above, a feature is recursively added to the chosen feature set based on supervised learning and the similarity measures. With the use of a classifier XXX, we call the first gene selection method XXX-MSC and the second one XXX-MMC. For example, if the classifier is Naive Bayes Classifier (NBC), we call the two strategies NBC-MSC and NBC-MMC, respectively.

### Lagging Prediction Peephole Optimization (LPPO)

We want to find a combination of features (genes) that yields the best performance on breaking down solvents. Normally, with the recursive addition for the next feature, the training accuracy will increase and reach a peak classification performance at some point, and then may maintain it with subsequent feature additions; but after that the training accuracy may decrease. Generally speaking, all strategies for determining the final feature set should be based on the best training classification. In high-volume data analysis, it is common that the best training accuracy corresponds to different feature sets; that is, multiple feature sets achieve the same highest training accuracy. However, although all these feature sets are associated with the same highest training accuracy, the testing accuracy of these feature sets may be different. Among these highest training feature sets, the one having the best testing accuracy is called the optimal feature set, which is highly complicated to characterize when a sample size is small. Either applying different gene methods to the same training samples, or applying the same gene selection method to different training samples, or applying different learning classifiers to the same training samples, will produce a different optimization of the feature set. Pochet *et al. *[29] presented a method of determining the optimal number of genes by means of a cross-validation procedure; the drawback of this method is that it actually utilizes whole data information, including training samples and testing samples.

How do we choose the optimal feature set? If there are multiple best training classifications, a random choice, called random strategy, works for best training classification. In the recursive addition of the features, for training samples, a classification model is one of the best methods. But for testing samples, at this point, the classification model may not be optimal because of the difference between the training samples and the testing samples; the optimal classification model will lag in appearance (see Figure 1). Based on this observation, we propose the following algorithm for optimization.

Under feature dimension *j*, the training accuracy of the *i*^th ^experiment is *r*(*i, j*). If the feature set **G***_k_*, corresponding to feature dimension *k*, has the best training accuracy in the trainings from the feature set **G**_1 _to **G**_D_, corresponding to the feature dimensions from 1 to D, let **HR **denote the set that contains all the combinations of **G***_k_*, corresponding to all the feature set having the highest classification accuracy under feature dimension 1 to D.

(11) $$HR=\left\{G_{k}|r\left(i,k\right)=\max\left(r\left(i,∙\right)\right),\;\;1\leq k\leq D\right\}$$

In general, the best classification model for testing samples will lag in appearance behind the initial best training model. We will exclude the elements of **HR **that correspond to the initial best training. The remaining elements in **HR **constitute the candidate set **HRC **for optimization.

Each element in **HRC **is associated with the best training accuracy. We set a peephole for each element and choose the element associated with the optimal peephole. The details are described as follows:

a. For each element **G***_k _*∈ **HRC**, the peephole over **G***_k _*with length of 2*l*+1 covers the feature sets **G***_k-l_*, **G**_*k-l+*1_, ..., **G***_k _*, ..., **G**_*k+l*-1_, **G***_k+l_*, corresponding to the training accuracy *r*(*i, k-l*), *r*(*i, k-l+*1), ..., *r*(*i, k*), ..., *r*(*i, k+l-*1), *r*(*i, k+l*). The mean training value of the peephole is denoted by mp_r(*i*, *k*).

(12) $$mp\text{\_}r\left(i,k\right)=\left(1∕\left(2l+1\right)\right)\sum_{m=k-l}^{m=k+l}r\left(i,m\right)$$

The peephole with the best classification of mp_r is then chosen as the optimal one.

b. If there are multiple optimal peepholes, then we apply random forest to these peepholes and check the mean values of the Out-of-Bag (OOB) error rates [24,25,30]. The feature sets **G***_k-l_*, **G**_*k-l*+1_, ..., **G**_*k*,_, ..., **G**_*k+l*-1, _**G***_k+l _*correspond to the OOB errors, oob_e(*i*, *k*-*l*), oob_e(*i*, *k*-*l+*1), ..., oob_e(*i*, *k*), ..., oob_e(*i*, *k*+*l-*1), oob_e(*i*, *k+l*). The mean value of the OOB errors is denoted by mp_oob_e(*i*, *k*)

(13) $$mp\text{\_}oob\text{\_}e\left(i,k\right)=\left(1∕\left(2l+1\right)\right)\sum_{m=k-l}^{m=k+l}oob\text{\_}e\left(i,m\right)$$

The peephole with minimum mp_oob_e is the optimal one.

c. If there are multiple peepholes corresponding to the best mp_r and minimum mp_oob_e, then set *l *+1 → *l*, and repeat 'a' to 'c', until a unique optimal peephole is determined.

d. The feature set located at the center of the final optimal peephole is chosen as the final optimal feature set.

**Figure 3:**
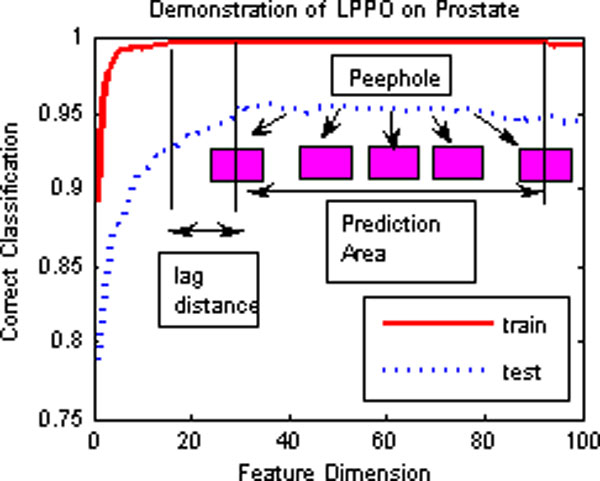
**A sketch description of the Lagging Prediction Peephole Optimization on Prostate data set**.

This optimization of RFA is called Lagging Prediction Peephole Optimization (LPPO). Figure 3 briefly outlines the LPPO on the prostate data set, which was studied by Singh *et a*l. [31].

### Data sets

The following six benchmark microarray data sets have been extensively studied and used in our experiments to compare the performances of our methods with others. Data sources that are not specified are available at: http://www.broad.mit.edu/cgi-bin/cancer/datasets.cgi.

1) The LEUKEMIA data set consists of two types of acute leukemia: 48 acute lymphoblastic leukemia (ALL) samples and 25 acute myeloblastic leukemia (AML) samples with over 7129 probes from 6817 human genes. It was studied by Golub *et al. *[32].

2) The LYMPHOMA data set consists of 58 diffuse large B-cell lymphoma (DLBCL) samples and 19 follicular lymphoma (FL) samples. It was studied by Shipp *et al. *[33]. The data file, lymphoma_8_lbc_fscc2_rn.res, and the class label file, lymphoma_8_lbc_fscc2.cls were used in our experiments for identifying DLBCL and FL.

3) The PROSTATE data set used by Singh *et al. *[31] contains 52 prostate tumor samples and 50 non-tumor prostate samples.

4) The COLON cancer data set used by Alon *et al. *[34] contains 62 samples collected from colon-cancer patients. Among them, 40 tumor biopsies are from tumors, and 22 normal biopsies are from healthy parts of the colons of the same patients. Based on the confidence in the measured expression levels, 2000 genes were selected. The data source is available at: http://microarray.princeton.edu/oncology/affydata/index.html.

5) The Central Nervous System (CNS) embryonal tumor data set that was originally studied by Pomeroy *et al. *[35] contains 60 patient samples. Among them, 21 are survivors who are alive after treatment, and 39 are failures who succumbed to their diseases. There are 7129 genes.

6) The Breast cancer data set studied by Van *et al. *[36] contains 97 patient samples, 46 of which are relapse patients who had developed distance metastases within 5 years, and 51 patients who are non-relapsed who remained healthy for at least 5 years from the distance after their initial diagnosis. This data source is available at: http://www.rii.com/publications/2002/vantveer.htm.

### Experiments

Our experiments are designed as follows:

1. The data sets are first divided randomly into training samples and testing samples. The ratio of training samples to testing samples is approximately 1:1 in each class.

2. Recursive feature additions with Naive Bayes Classifier (NBC) and Nearest Mean Scaled Classifier (NMSC) for gene selection (NBC-MSC, NBC-MMC, NMSC-MSC, and NMSC-MMC) were applied to the training samples for gene selection. Different feature sets of the gene expression data are produced under feature dimensions 1 to 100. We compared the above proposed methods to several recently developed and published gene selection methods: LOOCSFS, GLGS, SVMRFE, SFFS-LS bound, SFS-LS bound, and also T-TEST.

3. To compare different gene selection methods, the learning classifiers including NBC, NMSC, SVM [37,38], and Random Forest are applied to the testing samples.

4. The experiments were performed in 20 runs, and the average testing accuracies were compared to evaluate performance.

## Authors' contributions

QL designed the algorithms, performed the study, and wrote the draft; AHS supervised the study and revised the draft; ZC helped designing the algorithms and experiments, provided the statistical analysis, and co-drafted the manuscript; LC, JL, MQ, YP, ZW and HX participated in algorithm design, programming, and helped editing the draft; and YP coordinated the project and assisted the draft and data analyses

## Competing interests

The authors do not claim any confliction of interests.
